# Supplementary material for: Automated Classification of Cognitive Workload Levels Based on Psychophysiological and Behavioural Variables of Ex-Gaussian Distributional Features
Source: Brain Sci. 2022 Apr 23;12(5):542. doi: 10.3390/brainsci12050542 (PMC9138891; doi:10.3390/brainsci12050542)
Supplement: Supplementary file 1 [file brainsci-12-00542-s001.zip › brainsci-1617790-supplementary.pdf]

SUPPLEMENTARY MATERIAL

Table S1. The features belonging to the clusters of high and medium importance according to SVM with linear kernel.

Low cognitive workload	Medium cognitive workload	High cognitive workload
mu of blink number in 10 s<br>mu of saccade amplitude<br>mu of saccade number in 10 s<br>mu of single trial response time<br>tau of correct answers number in 10 s<br>tau of saccade amplitude<br>tau of single trial response time	mu of blink number in 10 s<br>mu of saccade amplitude<br>mu of saccade number in 10 s<br>mu of single trial response time<br>tau of correct answers number in 10 s<br>tau of fixation duration<br>tau of saccade amplitude<br>tau of single trial response times	mu of correct answers number in 10 s<br>tau of correct answers number in 10 s<br>tau of saccade duration<br>tau of saccade number in 10 s

Table S2. The features belonging to the clusters of high and medium importance according to Logistic Regression.

Low cognitive workload	Medium cognitive workload	High cognitive workload
mu of correct answers number in 10 s<br>mu of saccade amplitude<br>mu of saccade number in 10 s<br>mu of single trial response time<br>tau of correct answers number in 10 s<br>tau of fixation duration<br>tau of saccade amplitude	mu of blink number in 10 s<br>mu of saccade amplitude<br>mu of saccade number in 10 s<br>mu of single trial response time<br>tau of correct answers number in 10 s<br>tau of fixation duration<br>tau of saccade amplitude<br>tau of single trial response time	mu of correct answers number in 10 s<br>tau of correct answers number in 10 ss

Table S3. Separate class feature names with weights arranged according to the rankings obtained by interpreting the weights of the Logistic Regression model with elastic net regularization for cognitive features.

Feature number	Low	Medium	High
1	mu of single trial response time<br>1.51	tau of correct answers number in 10 s<br>1.90	tau of correct answers number in 10 s<br>2.47
2	tau of correct answers number in 10 s<br>0.90	mu of single trial response time<br>1.61	mu of correct answers number in 10 s<br>0.88
3	mu of correct answers number in 10 s<br>0.69	tau of single trial response time<br>0.50	tau of single trial response time<br>0.15
4	tau of single trial response time<br>0.30	mu of correct answers number in 10 s<br>0.39	mu of single trial response time<br>0.12

Table S4. Separate class feature names with weights arranged according to the rankings obtained by interpreting the weights of the Logistic Regression model with elastic net regularization for eye-tracking features.

Feature number	Low	Medium	High
1	mu of saccade amplitude<br>1.00	mu of saccade amplitude<br>0.77	tau of saccade number in 10 s<br>0.36
2	tau of saccade amplitude<br>0.90	mu of saccade number in 10 s<br>0.71	tau of saccade duration<br>0.29
3	mu of saccade number in 10 s<br>0.74	tau of saccade amplitude<br>0.68	tau of saccade amplitude<br>0.20
4	tau of fixation duration<br>0.58	tau of fixation duration<br>0.58	mu of fixation number in 10 s<br>0.18

5	mu of blink number in 10 s<br>0.52	mu of blink number in 10 s<br>0.48	tau of fixation number in 10 s<br>0.14
6	tau of saccade duration<br>0.33	mu of fixation number in 10 s<br>0.36	mu of fixation duration<br>0.14
7	mu of fixation number in 10 s<br>0.28	mu of fixation duration<br>0.25	mu of saccade amplitude<br>0.13
8	mu of fixation duration<br>0.22	tau of saccade number in 10 s<br>0.21	mu of saccade duration<br>0.12
9	tau of fixation number in 10 s<br>0.22	tau of fixation number in 10 s<br>0.19	tau of blink number in 10 s<br>0.09
10	tau of blink number in 10 s<br>0.19	tau of saccade duration<br>0.16	mu of blink number in 10 s<br>0.08
11	tau of saccade number in 10 s<br>0.15	mu of saccade duration<br>0.14	tau of fixation duration<br>0.06
12	mu of saccade duration<br>0.14	tau of blink number in 10 s<br>0.14	mu of saccade number in 10 s<br>0.05

Table S5. Separate class feature names with weights arranged according to the rankings obtained by interpreting the weights of the linear SVM model for cognitive features.

Feature number	Low	Medium	High
1	mu of single trial response time<br>1.21	tau of correct answers number in 10 s<br>1.59	tau of correct answers number in 10 s<br>1.24
2	tau of correct answers number in 10 s<br>0.43	mu of single trial response time<br>1.24	mu of correct answers number in 10 s<br>0.12
3	tau of single trial response time<br>0.37	tau of single trial response time<br>0.39	mu of single trial response time<br>0.07
4	mu of correct answers number in 10 s<br>0.24	mu of correct answers number in 10 s<br>0.25	tau of single trial response time<br>0.03

Table S6. Separate class feature names with weights arranged according to the rankings obtained by interpreting the weights of the linear SVM model for eye-tracking features.

Feature number	Low	Medium	High
1	mu of saccade amplitude<br>1.24	mu of saccade amplitude<br>1.18	tau of saccade number in 10 s<br>0.16
2	tau of saccade amplitude<br>1.12	tau of saccade amplitude<br>1.02	tau of saccade duration<br>0.15
3	mu of saccade number in 10 s<br>0.54	mu of saccade number in 10 s<br>0.64	tau of fixation number in 10 s<br>0.08
4	mu of blink number in 10 s<br>0.41	tau of fixation duration<br>0.35	tau of saccade amplitude<br>0.08
5	tau of fixation duration<br>0.31	mu of blink number in 10 s<br>0.33	mu of fixation number in 10 s<br>0.08
6	tau of saccade duration<br>0.28	mu of fixation number in 10 s<br>0.26	mu of blink number in 10 s<br>0.06
7	mu of fixation number in 10 s<br>0.25	mu of saccade duration<br>0.24	tau of blink number in 10 s<br>0.06
8	mu of saccade duration<br>0.21	tau of saccade duration<br>0.22	mu of saccade duration<br>0.05
9	mu of fixation duration<br>0.16	mu of fixation duration<br>0.20	mu of saccade amplitude<br>0.05
10	tau of blink number in 10 s<br>0.15	tau of blink number in 10 s<br>0.12	mu of saccade number in 10 s<br>0.05
11	tau of fixation number in 10 s<br>0.14	tau of fixation number in 10 s<br>0.12	mu of fixation duration<br>0.04
12	tau of saccade number in 10 s<br>0.13	tau of saccade number in 10 s<br>0.09	tau of fixation duration<br>0.03

Table S7. Ex-gaussian parameters describing the data used for classification

1	2	3	4	5	6	7	8	9	10	11	12	13	14	15	16	17	18
1	1	113.09	141.52	8.01	0.12	44.54	0.53	29.53	0.29	31.47	0.30	9.53	0.09	792.84	168.13	9.25	1.08
1	2	117.96	111.53	7.32	1.26	34.06	10.44	35.63	0.14	37.61	0.06	8.20	0.02	906.91	323.65	7.97	0.03
1	3	118.81	122.80	8.19	0.87	39.47	4.44	33.36	0.81	37.42	0.07	8.45	0.32	979.19	182.76	7.00	11.82
2	1	98.11	114.11	0.77	7.68	28.19	12.39	35.41	3.37	36.18	4.71	10.05	0.06	891.00	117.48	9.62	0.27
2	2	82.17	112.61	0.57	9.71	22.95	13.62	39.98	0.57	51.22	0.11	8.00	7.11	890.30	287.28	8.41	0.03
2	3	91.44	110.66	8.90	1.49	22.77	14.33	37.00	0.25	47.27	0.33	7.05	7.78	817.53	298.56	8.00	9.89
3	1	95.90	73.68	7.85	0.14	24.36	10.45	46.80	0.10	52.22	0.11	12.75	0.03	955.53	391.21	6.00	1.34
3	2	95.13	65.26	0.69	8.02	18.45	14.22	46.47	2.09	59.79	1.10	8.64	1.25	1141.93	190.59	7.00	0.44
3	3	90.14	71.95	0.94	8.04	17.07	14.33	45.75	2.14	60.75	0.45	11.18	0.04	993.23	212.85	2.93	11.97
4	1	110.29	113.43	10.30	0.17	34.99	7.10	32.00	5.19	37.31	0.39	9.68	0.11	482.22	326.36	11.27	0.95
4	2	123.40	83.16	9.80	0.17	38.47	0.56	34.60	0.32	42.44	0.39	8.51	0.07	667.45	435.42	9.00	0.04
4	3	127.95	79.82	9.75	0.17	39.47	0.52	36.48	0.25	43.42	0.32	9.41	0.07	615.56	354.29	3.25	15.25
5	1	95.36	153.78	8.16	0.14	45.32	0.55	29.86	0.28	31.37	0.29	9.08	0.03	686.95	312.74	9.95	0.06
5	2	91.74	152.28	6.80	2.49	39.11	7.79	33.58	0.08	32.74	2.81	6.34	1.33	885.37	298.76	8.41	0.04
5	3	106.22	156.26	7.73	2.20	48.40	0.64	29.47	0.24	31.57	0.23	7.11	0.44	806.47	246.04	4.03	13.07
6	1	164.31	70.05	8.92	0.13	39.17	7.42	31.50	0.29	32.97	0.31	10.00	1.00	871.84	295.61	8.53	0.02
6	2	142.12	90.67	9.87	0.73	41.55	8.00	33.93	1.18	36.17	0.05	8.71	0.85	1250.55	140.29	7.09	0.02
6	3	152.18	79.38	10.40	0.25	40.73	9.19	33.34	1.66	34.97	1.53	8.32	1.07	1043.55	361.43	2.61	10.19
7	1	102.55	119.30	7.02	1.65	35.48	6.21	34.72	1.84	37.71	1.16	10.22	0.06	761.71	263.18	9.22	0.74
7	2	102.08	104.97	5.04	4.33	30.61	9.33	38.05	0.64	44.76	0.89	7.94	1.96	898.39	307.14	8.16	0.12
7	3	107.09	96.31	6.95	2.64	29.70	8.40	38.15	0.86	47.22	0.29	9.03	2.05	851.38	262.11	5.30	12.23
8	1	107.44	87.80	7.80	0.15	29.67	6.67	39.85	1.04	44.90	0.11	11.17	0.05	714.16	193.03	10.97	0.03
8	2	116.18	70.64	0.69	8.77	25.41	10.38	41.15	1.74	49.78	0.10	8.48	0.85	871.10	294.20	8.53	0.02
8	3	112.87	82.19	7.15	2.54	28.12	7.37	41.13	0.09	45.94	3.17	9.29	0.04	967.41	170.05	3.27	12.53
9	1	95.92	103.58	9.17	0.15	26.30	12.16	36.26	0.09	54.12	0.21	45.47	35.08	586.91	522.50	8.96	0.05
9	2	104.10	121.30	9.27	1.31	33.99	9.84	35.46	0.08	36.00	5.35	9.00	2.43	907.00	320.04	8.09	0.02
9	3	125.82	107.79	10.02	1.39	34.67	11.06	34.32	0.06	39.36	0.08	9.45	2.44	900.85	282.28	2.21	12.99

10	1	98.69	113.57	6.61	0.12	27.99	13.36	37.88	0.12	41.19	0.13	11.51	0.05	696.91	512.09	8.03	0.20
10	2	94.77	102.04	0.50	7.53	26.52	16.81	39.32	0.12	44.07	0.14	8.14	3.19	773.15	532.21	6.50	0.94
10	3	98.05	98.10	0.52	8.73	20.31	24.99	39.83	0.11	42.76	0.13	7.34	3.83	982.15	284.51	7.00	8.56
11	1	99.54	145.09	7.40	0.13	39.36	0.58	34.14	0.09	35.19	0.34	8.00	1.23	930.63	214.37	8.64	0.03
11	2	99.28	122.96	0.73	7.69	16.73	22.69	36.38	0.06	43.95	1.16	5.00	7.89	1014.61	504.64	6.51	0.04
11	3	90.46	128.11	0.64	8.60	25.69	14.74	33.41	3.43	45.16	0.06	8.09	0.02	983.61	353.49	7.00	7.89
12	1	117.56	95.18	8.23	0.52	32.66	8.11	35.58	0.82	42.42	0.45	19.21	9.05	733.65	318.58	9.42	0.21
12	2	116.12	96.89	6.22	3.79	32.89	9.39	37.15	0.91	41.68	1.60	8.53	1.52	981.76	265.42	7.97	0.05
12	3	119.59	103.53	8.06	1.91	36.74	6.40	35.52	0.71	39.93	1.31	8.44	0.90	917.20	259.91	3.80	12.01
13	1	111.29	98.75	6.94	0.13	34.14	0.41	39.25	0.08	44.26	0.08	8.00	1.89	834.42	294.33	8.00	0.78
13	2	106.35	114.01	6.72	2.16	29.48	8.64	37.79	0.11	40.20	0.10	7.34	1.33	1000.88	280.71	7.55	0.23
13	3	113.29	88.67	7.82	0.94	28.47	9.41	40.44	0.06	44.37	0.08	8.47	0.47	676.96	521.51	2.97	11.93
14	1	103.42	100.95	9.07	0.15	39.57	0.42	37.00	3.11	42.33	0.12	10.39	1.84	715.14	172.65	10.24	0.98
14	2	102.74	72.23	6.08	3.48	36.57	4.31	40.91	0.39	42.81	0.40	8.52	0.08	947.57	158.57	8.98	0.02
14	3	93.96	104.89	8.85	1.41	35.71	4.61	39.49	0.79	41.92	0.34	8.99	0.07	823.36	315.25	2.80	13.00
15	1	117.94	120.61	8.02	0.14	25.19	8.77	35.10	0.12	41.79	0.10	8.00	1.67	924.58	271.62	7.25	1.08
15	2	124.96	114.07	9.58	0.18	30.72	6.69	34.59	0.08	36.25	3.53	7.00	1.73	1096.33	337.30	6.87	0.02
15	3	102.51	134.44	0.74	8.72	25.89	11.52	32.38	2.96	36.97	3.09	7.25	0.14	905.26	577.78	2.48	9.62
16	1	136.70	105.97	9.51	0.13	36.91	0.40	31.61	0.30	35.42	0.34	11.24	0.09	675.85	218.26	10.35	0.76
16	2	103.20	114.45	9.40	0.90	36.73	0.44	37.42	0.47	41.00	3.46	8.00	2.99	773.04	340.95	8.87	0.02
16	3	109.08	109.62	10.17	0.19	27.71	7.51	34.95	0.24	45.61	0.32	7.29	10.71	766.83	284.96	8.00	11.00
17	1	71.57	170.27	9.66	0.16	3.00	28.69	25.68	0.32	44.71	6.18	104.16	1.38	786.05	221.93	9.69	0.20
17	2	62.94	138.08	11.19	0.20	2.97	30.97	30.51	2.16	69.38	0.19	47.66	34.56	938.84	233.56	8.42	0.03
17	3	84.09	161.85	11.96	0.20	3.00	29.38	27.84	0.20	58.68	0.43	65.59	0.62	905.94	283.58	7.00	9.78
18	1	102.84	93.08	7.92	0.14	34.05	5.85	41.71	0.07	43.70	0.08	10.00	0.85	702.70	359.61	9.29	0.04
18	2	103.48	77.74	0.79	7.94	24.58	15.79	44.04	0.08	47.26	0.08	7.00	4.51	893.83	505.17	6.35	0.76
18	3	102.00	76.42	0.70	8.39	27.37	12.47	44.46	0.32	48.47	0.08	8.43	0.57	947.88	219.35	2.84	12.56
19	1	107.87	171.84	8.56	0.13	39.40	0.38	28.00	3.87	29.17	2.27	11.19	0.03	733.80	195.71	10.11	0.56
19	2	115.11	126.65	8.56	0.74	39.11	0.43	35.05	0.06	36.27	0.07	7.87	0.91	990.44	199.04	8.00	0.74
19	3	110.91	136.68	9.27	0.37	39.57	0.44	34.24	0.21	33.86	0.24	8.26	1.08	873.59	242.15	3.44	12.66

20	1	114.14	126.16	8.23	0.12	25.06	9.02	30.82	0.30	37.06	0.35	13.87	0.13	616.71	246.45	11.53	0.02
20	2	110.24	82.25	8.42	0.45	25.92	7.90	38.45	2.77	46.80	0.09	11.39	2.39	711.93	309.26	9.52	0.14
20	3	114.69	80.72	8.80	1.33	29.96	8.28	38.30	0.31	43.09	0.32	13.06	0.11	698.51	256.22	1.00	17.80
21	1	125.06	116.63	7.95	0.14	34.92	1.05	33.35	0.10	35.61	4.17	12.38	0.07	776.41	429.44	8.19	0.04
21	2	144.37	119.49	9.64	0.46	39.24	0.59	28.12	0.27	33.48	0.34	8.60	0.08	993.93	238.50	8.09	0.02
21	3	169.06	99.76	10.28	0.47	37.20	5.58	29.35	0.20	33.14	0.23	8.59	1.19	849.45	241.27	2.99	13.51
23	1	106.77	113.15	7.29	0.23	33.54	5.62	36.71	0.28	40.76	0.25	11.68	3.06	798.90	334.84	8.52	0.30
23	2	103.39	107.30	2.48	6.33	25.38	16.30	37.62	0.36	43.23	0.97	7.23	4.20	923.17	434.09	6.99	0.34
23	3	108.48	109.38	4.81	5.16	29.35	14.30	35.77	1.08	41.80	0.39	8.33	1.80	945.95	295.05	5.00	10.36
24	1	113.82	170.36	9.77	0.12	34.91	1.67	25.78	0.25	41.22	0.39	9.37	1.85	713.28	304.92	9.63	0.03
24	2	102.37	102.00	8.64	3.05	16.15	23.55	36.03	0.86	52.00	5.89	11.00	4.08	879.15	394.70	7.74	0.03
24	3	99.38	111.73	12.72	0.19	10.07	33.06	32.94	0.27	51.92	0.37	31.78	22.53	838.40	200.32	3.52	13.78
25	1	92.83	100.51	8.50	0.15	3.00	34.89	38.53	0.14	54.00	6.35	109.21	1.69	752.30	201.36	10.42	0.03
25	2	111.57	54.18	6.55	4.29	3.00	38.91	38.90	0.36	56.90	0.56	46.75	10.36	844.31	226.33	8.26	1.08
25	3	97.68	84.69	9.16	1.87	31.48	8.89	37.00	0.30	57.31	0.44	60.63	0.71	820.85	183.58	9.00	10.63
26	1	136.98	137.69	9.05	0.13	32.95	1.10	27.69	0.24	38.94	0.36	9.62	0.09	711.51	277.48	9.00	1.33
26	2	123.05	125.08	8.16	0.85	4.62	27.30	33.79	0.10	37.31	9.58	8.06	0.05	761.53	405.34	8.50	0.04
26	3	127.60	123.14	8.20	1.33	30.12	4.42	31.24	0.25	41.75	0.32	7.72	1.16	711.27	298.95	1.00	16.49
27	1	73.14	114.97	0.47	6.22	25.71	8.72	39.19	0.38	41.30	0.39	9.98	0.10	636.14	373.30	9.82	0.07
27	2	85.01	90.99	0.42	7.30	14.77	18.62	45.74	0.03	50.51	0.38	10.19	0.03	1025.71	242.57	7.55	0.23
27	3	83.61	92.67	0.46	7.64	13.39	20.75	42.34	0.34	47.89	0.38	10.03	0.07	931.78	247.24	7.00	9.89
28	1	70.00	136.33	7.92	0.14	3.00	27.30	35.57	0.10	68.28	0.16	11.00	3.23	774.27	162.63	9.39	1.28
28	2	60.00	131.77	0.61	8.29	3.00	28.33	38.67	1.11	67.22	0.11	10.09	0.14	864.39	255.87	8.86	0.03
28	3	61.17	127.11	6.80	1.96	3.00	29.76	36.11	0.29	68.56	0.55	9.26	1.40	904.96	216.33	8.00	9.78
29	1	104.83	170.50	8.48	0.14	39.33	0.45	28.31	0.29	29.20	0.30	9.62	0.27	756.69	265.28	8.00	2.01
29	2	103.45	136.40	9.56	0.16	34.05	2.70	34.60	0.07	42.59	0.10	7.77	0.12	955.26	326.25	7.75	0.03
29	3	109.67	135.76	9.52	0.16	25.94	11.79	32.00	0.22	39.10	0.27	7.34	1.05	1021.71	254.85	2.54	11.56
30	1	84.75	155.64	7.64	0.11	39.26	0.51	28.40	5.93	35.00	4.89	10.07	0.04	664.80	343.82	9.85	0.04
30	2	96.74	127.04	7.10	2.65	39.75	0.58	35.58	0.08	45.17	0.17	7.00	4.06	795.21	229.96	3.74	12.96
30	3	109.51	101.74	9.31	0.97	45.09	0.46	37.94	0.23	38.80	2.42	8.73	1.50	795.58	357.94	8.63	0.03

Legend of the table header:

1. patient id
2. cognitive workload level
3. mu of fixation duration
4. tau of fixation duration
5. mu of saccade amplitude
6. tau of saccade amplitude
7. mu of saccade duration
8. tau of saccade duration
9. mu of fixation number in 10 s
10. tau of fixation number in 10 s
11. mu of saccade number in 10 s
12. tau of saccade number in 10 s
13. mu of blink number in 10 s
14. tau of blink number in 10 s
15. mu of single trial response time
16. tau of single trial response time
17. mu of correct answers number in 10 s

tau of correct answers number in 10 s
